# Tumor Suppressive Role of the *PRELP* Gene in Ovarian Clear Cell Carcinoma

**DOI:** 10.3390/jpm12121999

**Published:** 2022-12-02

**Authors:** Ai Dozen, Kanto Shozu, Norio Shinkai, Noriko Ikawa, Rina Aoyama, Hidenori Machino, Ken Asada, Hiroshi Yoshida, Tomoyasu Kato, Ryuji Hamamoto, Syuzo Kaneko, Masaaki Komatsu

**Affiliations:** 1 Division of Medical AI Research and Development, National Cancer Center Research Institute, 5-1-1 Tsukiji, Chuo-ku, Tokyo 104-0045, Japan; 2 Department of Obstetrics and Gynecology, Keio University School of Medicine, 35 Shinanomachi, Shinjuku-ku, Tokyo 160-8582, Japan; 3 Department of Obstetrics and Gynecology, University of Toyama, 2630 Sugitani, Toyama 930-0194, Japan; 4 Cancer Translational Research Team, RIKEN Center for Advanced Intelligence Project, 1-4-1 Nihonbashi, Chuo-ku, Tokyo 103-0027, Japan; 5 Department of NCC Cancer Science, Biomedical Science and Engineering Track, Graduate School of Medical and Dental Sciences, Tokyo Medical and Dental University, 1-5-45 Yushima, Bunkyo-ku, Tokyo 113-8510, Japan; 6 Department of Obstetrics and Gynecology, Showa University School of Medicine, 1-5-8 Hatanodai, Shinagawa-ku, Tokyo 142-8666, Japan; 7 Department of Diagnostic Pathology, National Cancer Center Hospital, 5-1-1 Tsukiji, Chuo-ku, Tokyo 104–0045, Japan; 8 Department of Gynecology, National Cancer Center Hospital, 5-1-1 Tsukiji, Chuo-ku, Tokyo 104–0045, Japan

**Keywords:** ovarian clear cell carcinoma, PRELP, gene expression, epigenetics

## Abstract

Ovarian clear cell carcinoma (OCCC) has a poor prognosis, and its therapeutic strategy has not been established. PRELP is a leucine-rich repeat protein in the extracellular matrix of connective tissues. Although PRELP anchors the basement membrane to the connective tissue and is absent in most epithelial cancers, much remains unknown regarding its function as a regulator of ligand-mediated signaling pathways. Here, we obtained sets of differentially expressed genes by PRELP expression using OCCC cell lines. We found that more than 1000 genes were significantly altered by PRELP expression, particularly affecting the expression of a group of genes involved in the PI3K-AKT signaling pathway. Furthermore, we revealed the loss of active histone marks on the loci of the *PRELP* gene in patients with OCCC and how its forced expression inhibited cell proliferation. These findings suggest that PRELP is not only a molecule anchored in connective tissues but is also a signaling molecule acting in a tumor-suppressive manner. It can serve as the basis for early detection and novel therapeutic approaches for OCCC toward precision medicine.

## 1. Introduction

Ovarian cancer is a leading cause of gynecological cancer mortality worldwide [1]. In the early stages, only a few subjective symptoms are present, and no screening method for ovarian cancer has been established. Even if patients with ovarian cancer initially respond favorably to first-line platinum-based chemotherapy, it is estimated that ≥80% of these patients will eventually relapse [2]. Although several target therapies have been applied for ovarian cancer, including poly (ADP-ribose) polymerase (PARP) inhibitors and vascular endothelial growth factor (VEGF) inhibitors, a selectable therapeutic approach for relapse or treatment resistance remains limited [3]. For the early detection of ovarian cancer and to develop novel therapeutic approaches toward precision medicine, further investigation of the molecular mechanisms underlying ovarian tumorigenesis and progression is required.

Epithelial ovarian cancers comprise five main histological subtypes: high-grade serous, endometrioid, clear cell, mucinous, and low-grade serous carcinomas. High-grade serous ovarian cancer (HGSOC) is the most common type of ovarian cancer. Accumulating pathological, epidemiological, and molecular evidence has revealed that fallopian tube secretory epithelial cells are the likely progenitors of HGSOC [4,5]. Except for *TP53, BRCA1*, and *BRCA2*, point mutations in oncogenes or tumor suppressor genes are relatively uncommon in HGSOC. Instead, several chromosomal structural variations have been reported in HGSOC [6]. Approximately 50% of HGSOC cases show defects in the homologous recombination (HR) DNA repair pathway, which is a key determinant of platinum sensitivity and provides a rationale for the use of PARP inhibitors [7,8]. In contrast, ovarian clear cell carcinoma (OCCC) accounts for approximately 10% of all ovarian cancers. Endometriosis is reported to be a risk factor for OCCC and coexists in more than 50% of cases [9,10]. Although OCCC is estimated to originate from ovarian endometriotic epithelial cells, the exact mechanism of its tumorigenesis has not been fully elucidated and awaits further study. OCCC shows a distinctive molecular pathogenetic pathway and intrinsic chemoresistance, which makes this entity unique compared to the other subtypes [11,12]. However, a therapeutic strategy specific to OCCC has not yet been established due to its relative rarity, and OCCC is usually treated in the same way as HGSOC. Patients with advanced or recurrent OCCC experience poorer clinical outcomes compared to those with HGSOC.

Small leucine-rich proteoglycans (SLRPs) are a family of 17 known proteoglycans, which are secreted proteins in the extracellular matrix (ECM). Regarding their function, SLRPs not only modify ECM organization but also serve as regulators of ligand-mediated signaling pathways [13,14,15,16]. Using a public dataset, we have previously shown that the *PRELP* gene, one of the secreted ECMs, is markedly downregulated in most epithelial cancers. Moreover, its ectopic expression in bladder cancer cell lines inhibited the transforming growth factor-beta and the epidermal growth factor pathways, induced cell–cell adhesion, and reversed epithelial–mesenchymal transition (EMT) [17].

In this study, we aimed to investigate the molecular mechanisms underlying OCCC tumorigenesis toward the early detection and novel therapeutic strategy for OCCC. We performed genetic and epigenetic analyses of the *PRELP* gene using OCCC cell lines and clinical samples. To our knowledge, this is the first study to focus on the *PRELP* gene in OCCC.

## 2. Materials and Methods

### 2.1. Clinical Materials

Both normal ovarian tissues and tumor tissues of the same patient were collected during primary debulking surgery. Fresh-frozen samples of OCCC (*n* = 7) were obtained from collections at the University of Tokyo Hospital and the National Cancer Center Hospital (NCCH). Fresh-frozen samples of HGSOC (*n* = 1), low-grade serous ovarian carcinoma (*n* = 1), and carcinosarcoma (*n* = 1) were obtained from collections at the NCCH (Supplementary Table S1). The clinical specimens were stored at –80 °C and embedded into the OCT compound, followed by frozen sectioning and RNA extraction. The histology was determined based on the pathologist’s assessment according to the 2020 World Health Organization classification of female genital tumors.

### 2.2. Database Analysis

To compare the expression of tumor and normal tissues in the ovary, we reanalyzed using the same pipeline of RNA sequencing (RNA-seq) from The Cancer Genome Atlas (TCGA) and the Genotype-Tissue Expression project datasets on the UCSC Xena platform (http://xena.ucsc.edu/, accessed on 2 May 2022) [18]. RNA-seq, DNA copy number, gene mutation, and clinical data of 316 patients with HGSOC in the TCGA cohort were sourced from the cBioPortal for Cancer Genomics (http://www.cbioportal.org/, accessed on 2 May 2022) [19,20,21]. The individual data used to generate the graphs are listed in Supplementary Tables S2–S4.

### 2.3. Cell Culture

Human embryonic kidney cells expressing SV40 large T antigen 293T and the ovarian cancer cell line ES2 were cultured as described previously [22,23]. The immortalized human ovarian endometriotic epithelial cell line HMOsisEC10 was grown in an F-medium supplemented with 5% fetal bovine serum (FBS) (10270106, Thermo-Fisher Scientific, Waltham, MA, USA) and 1% antibiotic-antimycotic (100×; 15240-062, Thermo-Fisher Scientific, Waltham, MA, USA) [24]. The ovarian cancer cell line SKOV3 (HTB-77) was purchased from the American Type Culture Collection (Manassas, VA, USA). OVISE (JCRB1043), OVTOKO (JCRB1048), and RMG-I (JCRB0172) cell lines were purchased from the Japanese Collection of Research Biosources Cell Bank (JCRB Cell Bank, Osaka, Japan). OVISE and OVTOKO cells were maintained in the Roswell Park Memorial Institute-1640 (189-02025, FUJIFILM Wako Pure Chemical Co., Osaka, Japan) with 10% FBS and 1% antibiotic-antimycotic. RMG-I cells were maintained in Ham’s F-12 (087-08335, FUJIFILM Wako Pure Chemical Co., Osaka, Japan) with 10% FBS and 1% antibiotic-antimycotic. SKOV3 cells were cultured in McCoy’s 5A (16600082, Thermo-Fisher Scientific, Waltham, MA, USA) with 10% FBS and 1% antibiotic-antimycotic. PRELP-myc expressed cells were cultured as described above, except that we used Tet-system-approved FBS (631101, Takara Bio, Inc., Shiga, Japan). The establishment of a conditional protein expression system has been essentially described [22,23]. All cell lines were certified by STR profiling cell line authentication as described in Supplementary Table S11. Mycoplasma negative testing was performed using the e-Myco Mycoplasma PCR Detection Kit (25235, iNtRON Biotechnology, Inc., Seongnam, Korea).

### 2.4. Reverse Transcription Polymerase Chain Reaction (RT-PCR)

The general RT-PCR procedure was performed as reported previously [22,23]. PRELP mRNA levels were normalized to glyceraldehyde 3-phosphate dehydrogenase (GAPDH) mRNA levels as an internal control using the ΔCq method. For quantitative real-time PCR in triplicate for each sample, we used the following primers: PRELP_Forward: 5′-CTG TCC CAC AAC AGG ATC AG-3′; PRELP_Reverse: 5′-CAG GTC CGA GGA GAA GTC AT-3′; GAPDH_Forward: 5-GCA AAT TCC ATG GCA CCG TC-3′; GAPDH_Reverse: 5′-TCG CCC CAC TTG ATT TTG G-3′.

### 2.5. Plasmids

The procedure for plasmid construction was performed as reported previously [22,23]. The PRELP-myc cDNA was PCR amplified with the following primers—PRELP-myc_F_NheI: 5′-ACC CAA GCT GGC TAG CCA CCA TGA GGT CAC CCC TCT GCT G -3′, PRELP-myc_R_NotI: 5′-CAG CAC AGT GGC GGC CGC TCG AGT CTA GAC TAT AGT TCT AGA GGC TCG A-3′—and cloned into the modified Edit-R Inducible Lentiviral Plasmid at the NheI and NotI sites. All plasmids were verified by Sanger sequencing.

### 2.6. Western Blotting

The general procedure for Western blotting was performed as reported previously [22,23]. The following antibodies were used: anti-myc (sc-40; 1:1000 dilution, Santa Cruz Biotechnology, Dallas, TX, USA); alpha-tubulin (CP06; 1:1000 dilution, Merck Millipore, Darmstadt, Germany); anti-mouse IgG (NA931; 1:5000 dilution, GE Healthcare, Chicago, IL, USA); and anti-rabbit IgG (NA934; 1:5000 dilution, GE Healthcare, Chicago, IL, USA).

### 2.7. Cell Viability Assay

The general procedure for the cell viability assay was performed as reported previously [22,23]. Conditionally PRELP-expressing cells were plated in 96-well plates at the following concentrations: 1500 cells/well for RMG-I and 1000 cells/well for OVTOKO and SKOV3. At the indicated time, 10 μL of the Cell Counting Kit-8 (343-07623, Dojindo, Kumamoto, Japan) reagent was added to each well. Cell viability was measured by detecting the absorbance at 450 nm using Multiskan FC (Thermo-Fisher Scientific, Waltham, MA, USA).

### 2.8. Soft Agar Colony Formation Assay

We performed a soft agar colony formation assay, which is an anchorage-independent growth assay, as described previously [25]. Briefly, twice the usual amount of serum and antibiotics was added to this solution and was mixed with the 1.2% SeaPlaque agarose solution (50100, Lonza, Basel, Switzerland) to yield a final 1× growth medium with 0.6% agarose solution. A sterile solution of 0.7% SeaPlaque agarose was mixed with the aforementioned 2× growth medium to yield 1× growth medium with 0.35% agarose. A suspension of 5000 cells was added to a 1.5 mL aliquot of the 1× growth medium with 0.35% agarose solution, and the resultant mixture was plated out in a 6-well dish on top of the 1× growth medium with a 0.6% agarose layer, which was prepared in advance. The plates were incubated for approximately 2–3 weeks until visible colonies appeared. The colonies were stained with 1-mg/mL p-nitroblue tetrazolium chloride (144-01993, FUJIFILM Wako Pure Chemical Co., Osaka, Japan) for 18 h. Phase-contrast images were acquired using Celldiscoverer 7 (ZEISS, Oberkochen, Germany) and analyzed using ImageJ (National Institutes of Health, MD, USA) [26].

### 2.9. Chromatin Immunoprecipitation Followed by Sequencing (ChIP-Seq)

The ChIP-seq procedure for frozen tissues was performed as described previously [27]. ChIP antibodies for H3K4me3 (9727, lot# 5, Cell Signaling Technology, Denver, CO, USA), H3K27ac (ab4729, lot# GR321673-1, Abcam, Cambridge, UK), CTCF (3418, lot# 3, Cell Signaling Technology, Denver, CO, USA), and H3K27me3 (9733, lot# 8, Cell Signaling Technology, Denver, CO, USA) were added to the sheared chromatin (10 μg for CTCF and approximately 250–500 ng for the modified histones), and the mixture was incubated in an ultrasonic water bath for 30 min at 4 °C. After centrifugation, the supernatants were incubated with FG Beads HM Protein G (TAB8848N3173, Tamagawa Seiki Co., Ltd., Nagano, Japan) for 30 min at 4 °C. The beads were washed twice with the ChIP buffer and washed with the Wash buffer as described previously [27]. Immunoprecipitated chromatin was eluted and reverse-crosslinked according to the manufacturer’s instructions (9003, Cell Signaling Technology, Denver, CO, USA). Immunoprecipitated DNA was purified using QIAquick PCR Purification Kit (28106, QIAGEN, Venlo, Netherlands). DNA libraries were prepared using the Accel-NGS 2S Plus DNA Library kit (21096, Swift Biosciences, Inc., Ann Arbor, MI, USA). The DNA libraries were quantified, and their size was determined using Agilent 2100 Bioanalyzer. DNA libraries were sequenced on Illumina sequencers (Illumina HiSeq 3000; Illumina, Inc., San Diego, CA, USA).

### 2.10. RNA-Seq

Conditionally PRELP-expressing cells derived from the OVTOKO and SKOV3 cell lines were treated with 1-μg/mL DOX for 72 h. The cells were washed with ice-cold PBS (-), and the total RNA was extracted using QIAzol Lysis Reagent and RNeasy Plus Mini Kit (73404, Qiagen, Venlo, Netherlands), according to the manufacturer’s instructions. Oligo (dT)-conjugated beads were used to isolate mRNA. After mixing with the fragmentation buffer, mRNA was fragmented, and cDNA was synthesized using the mRNA fragments as templates. Short DNA fragments were purified and resolved with elution buffer for end reparation. Subsequently, the short DNA fragments were ligated with adapters. The suitable fragments were selected for the PCR amplification as templates. During the QC steps, Agilent 2100 Bioanalyzer was used to quantify the sample library. Finally, the DNA libraries were sequenced using BGISEQ-500.

### 2.11. Bioinformatic and Statistical Analysis

The sequenced reads from the RNA-seq and ChIP-seq experiments were mapped to the hg38 version of the human genome with Bowtie2 (v2.2.9) and parameters –local [28]. For RNA-seq, counting reads were obtained using featureCounts (v1.5.0) with Homo_sapiens. GRCh37.75.gtf. Differential expression analysis was performed using edgeR (v3.18.1). Gene ontology and pathway analysis was performed using the clusterProfiler R package (v3.14.3). For the volcano plot representation, we used the EnhancedVolcano (v1.4.0) package. For ChIP-seq, normalized enriched regions were visualized using the Integrative Genomics Viewer, IGV (v2.3.91). All statistical analyses were performed using GraphPad Prism (v7.0.0; GraphPad Software, San Diego, CA, USA) and R (https://www.R-project.org/, accessed on 8 April 2021). *p*–values are indicated in the figures and figure legends.

## 3. Results

### 3.1. PRELP Gene Expression and Genomic Aberrations

**Figure 1:**
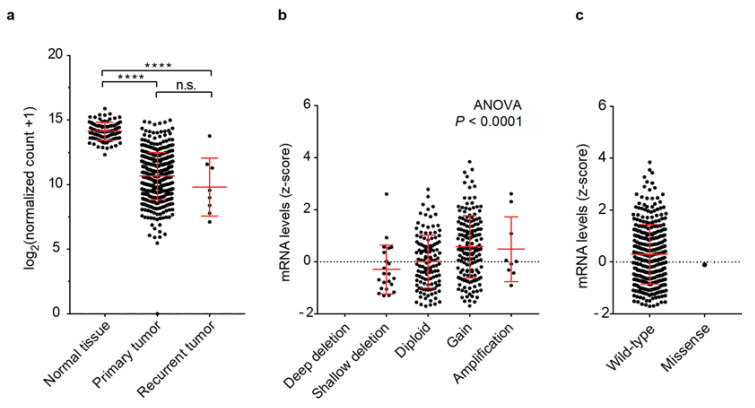
Correlation between *PRELP* gene expression and genetic mutation and copy number alteration (CNA) in ovarian cancer. (**a**) *PRELP* mRNA expression (y-axis) is plotted against normal tissue (*n* = 88), primary tumor (*n* = 419), recurrent tumor (*n* = 8). Statistical analysis was performed using Student’s *t*-test. **** *p* < 0.0001, n.s.: not significant. (**b**) *PRELP* mRNA expression (z-scores relative to diploid samples (y-axis) is plotted against CNAs in the *PRELP* gene (*n* = 316). Shallow deletion (CNA = −1), diploid (CNA = 0), gain (CNA = +1), and amplification (CNA = +2) are shown. (**c**) *PRELP* mRNA expression (z-scores relative to diploid samples; y-axis) is plotted against a wild-type (*n* = 315) or missense mutation (*n* = 1; x-axis).

To investigate the association between the *PRELP* mRNA expression and genomic aberrations, we first analyzed the correlation between *PRELP* gene expression and somatic mutations and copy number aberrations (CNAs) using a comprehensive genomic dataset of patients with ovarian cancer (see Materials and Methods). We found that *PRELP* mRNA expression was significantly repressed in both primary and recurrent ovarian cancers compared with that in normal tissues (*p* < 0.0001) (Figure 1a and Supplementary Table S2). Deletions in the *PRELP* gene were found in 6.9% of the cases, and the *PRELP* gene was amplified in 53.3% of the cases. These alterations were moderately correlated with *PRELP* mRNA expression (Figure 1b and Supplementary Table S3). PRELP retained its wild-type form in 99.7% of the cases (Figure 1c and Supplementary Table S4). These results suggest that *PRELP* mRNA expression is suppressed in ovarian cancer, which is not dependent on genetic deletion and mutation but rather epigenetic mechanisms, such as DNA methylation or histone modification.

**Figure 2:**
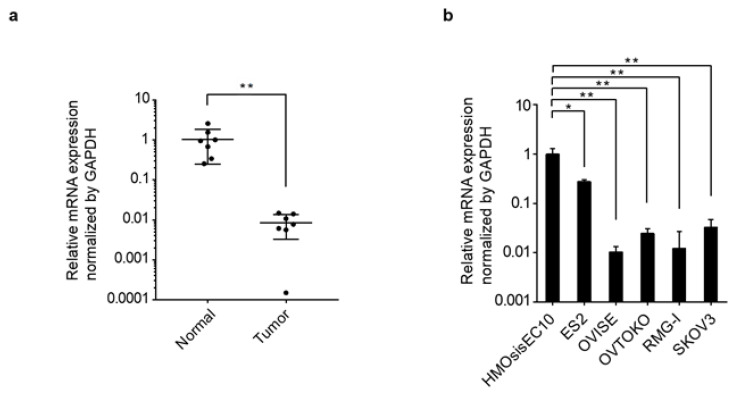
*PRELP* gene expression in ovarian cancer. (**a**) RT-PCR analysis using ovarian cancer tissue and matched normal ovarian tissue as a comparison. (**b**) RT-PCR analysis using HMOsisEC10, an immortalized human ovarian endometriotic epithelial cell line, and ovarian clear cell carcinoma (OCCC) cell lines. Relative mRNA expression is shown. RT-PCR was performed in triplicate for each sample. Statistical analysis was performed using Student’s *t*-test. * *p* < 0.05; ** *p* < 0.01.

Next, we evaluated *PRELP* gene expression using clinical tissues that matched with those from the patients with ovarian cancer, including OCCC, HGSOC, low-grade serous carcinoma, and carcinosarcoma. *PRELP* mRNA expression was significantly repressed in ovarian cancer tissues by approximately 1/100th compared with that in normal tissues (*p* < 0.01) (Figure 2a). Additionally, we compared *PRELP* gene expression between an immortalized human ovarian endometriotic epithelial cell line (HMOsisEC10) and OCCC cell lines, including ES2, OVISE, OVTOKO, RMG-I, and SKOV3. All OCCC cell lines showed a significantly reduced mRNA expression in *PRELP* compared with HMOsisEC10 (Figure 2b). These results indicate that the expression of *PRELP* was drastically altered in all the clinical specimens and cell lines we examined.

### 3.2. Cell Viability upon PRELP Expression

**Figure 3:**
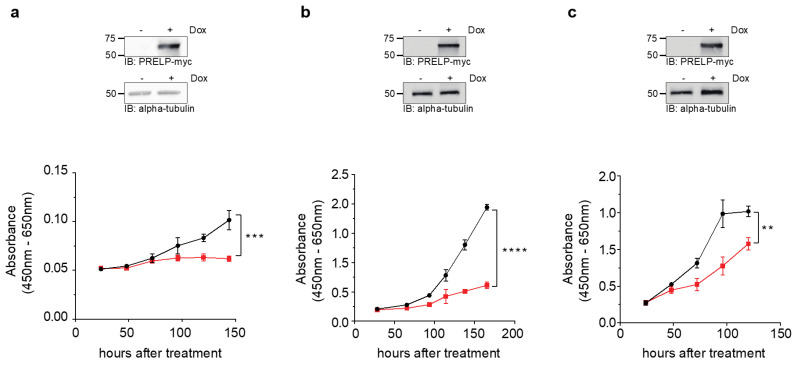
Reduced cell viability associated with induced *PRELP* gene expression in ovarian clear cell carcinoma (OCCC) cell lines. *PRELP* gene expression was induced by adding doxycycline (DOX) (1 μg/mL) to the lenti-viral expression system. The expression of myc-tagged PRELP protein was analyzed using whole-cell extracts from (**a**) RMG-I, (**b**) OVTOKO, and (**c**) SKOV3 cells with or without DOX. The left side indicates the protein size marker. Alpha-tubulin; loading control. The cell viability of (**a**) RMG-I, (**b**) OVTOKO, and (**c**) SKOV3 cells was evaluated using Cell-Counting Kit-8 assays. Black lines; without DOX, red lines; with DOX. Error bars indicate biological replicates (*n* = 3 or 4). Statistical analysis was performed using Student’s *t*-test. ** *p* < 0.01; *** *p* < 0.001; **** *p* < 0.0001.

To investigate the effects of *PRELP* expression on cell viability in OCCC, we stably introduced the *PRELP* gene into the RMG-I, OVTOKO, and SKOV3 cell lines. The transduced *PRELP* gene contained an inducible promoter (Tet-On system), which allowed us to control the timing of the *PRELP* gene expression by adding DOX to the cultured medium. Western blotting confirmed that the PRELP protein was expressed following the addition of DOX in all the cell lines (Figure 3, upper side). Under this condition, cell proliferation was significantly inhibited by PRELP expression (Figure 3, bottom side).

The soft agar colony formation assay with or without DOX treatment in *PRELP*-inducible SKOV3 showed that the induction of *PRELP* reduced the colonies (Supplementary Figure S1), suggesting the requirement of *PRELP* suppression for anchorage-independent growth. Note that the other *PRELP*-inducible RMG-I and OVTOKO cell lines could not be evaluated because they did not form colonies with or without DOX treatment.

### 3.3. ChIP-Seq in Clinical Tissues of OCCC

**Figure 4:**
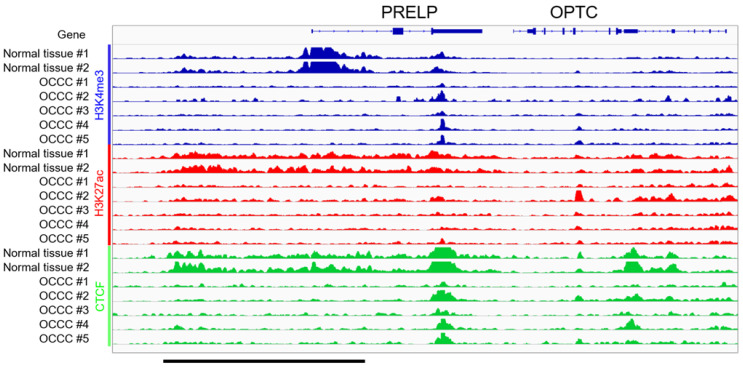
Loss of epigenetic marks at the *PRELP* locus in clinical tissues of ovarian clear cell carcinomas (OCCC). The integrative genomics viewer (IGV) tracks of H3K4me3 (blue), H3K27ac (red), and CTCF (green) peak at the *PRELP* locus, illustrating the loss of epigenetic marks around the *PRELP* gene promoter upstream region in OCCC (*n* = 5). Normal ovarian tissues (*n* = 2) on the opposite healthy ovary were used as a comparison. All data ranges are standardized as 0–2. The upstream region of the *PRELP* gene promoter is indicated by a black underline at the bottom of the figure.

The aforementioned results show that *PRELP* suppression in OCCC is likely mediated by epigenetic mechanisms, such as DNA methylation or histone modification. Given that there were no obvious CpG islands in the *PRELP* promoter region in the UCSC genome browser [29], we considered that an alteration of histone modifications might be involved in *PRELP* suppression. To this end, we performed a series of ChIP-seq on frozen normal ovarian tissues (*n* = 2) and OCCC tissues (*n* = 5) to investigate whether or not PRELP suppression was caused by the loss of active marks or the gain of suppressive marks. To investigate the molecular mechanisms underlying OCCC tumorigenesis, normal ovarian tissues with the same genetic background as OCCC tissues need to be analyzed. Therefore, normal ovarian tissues were collected from the opposite healthy ovaries of the same patients to avoid genetic and epigenetic alteration in those regions by inflammation and compression. The pathologist confirmed the histology of the normal ovarian tissues obtained. We found a loss in the active marks H3K4me3 and H3K27ac peaks around the upstream region of the *PRELP* gene in OCCC (Figure 4, blue and red). Interestingly, we also noticed the loss of CTCF in OCCC, which might reflect the promoter-proximal CTCF binding mediated by distal enhancer-dependent gene activation (Figure 4, green) [30]. In contrast, we did not observe a noticeable gain of the suppressive mark H3K27me3 in OCCC (Supplementary Figure S2). According to these results of our clinical ChIP-seq analyses using normal ovary and OCCC tissues, we can suggest that the loss of active histone markers in the promoter region of *PRELP* results in the loss of *PRELP* expression during tumorigenesis in OCCC.

### 3.4. Gene Sets Altered by the Overexpression of PRELP in OCCC Cell Lines

**Figure 5:**
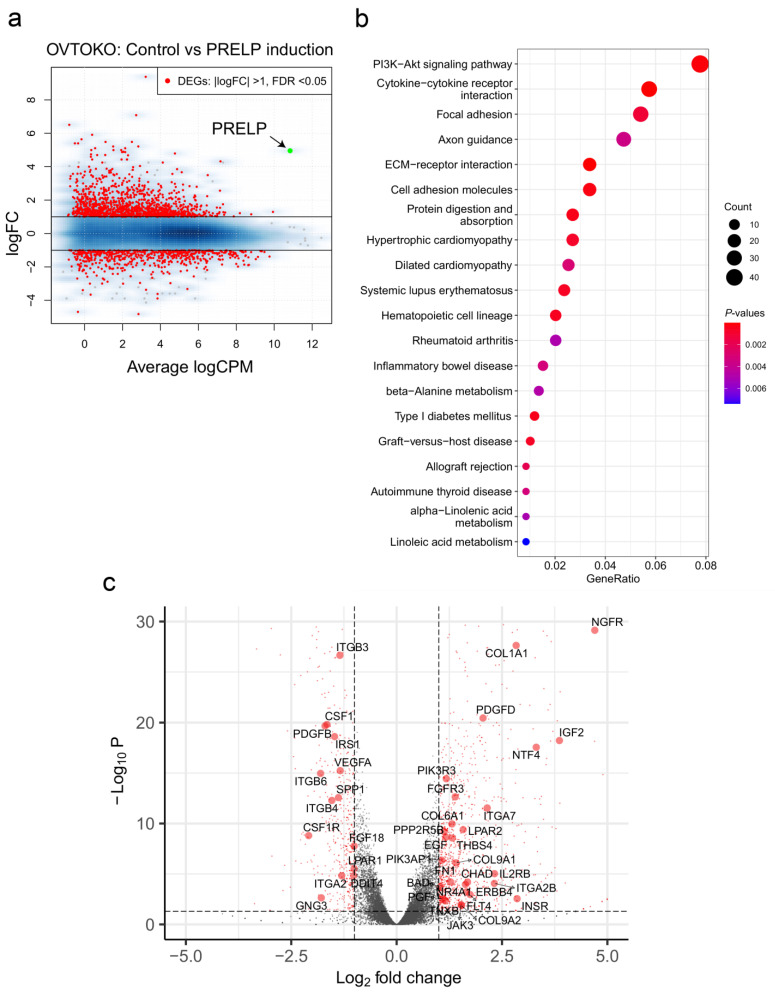
PRELP regulates the PI3K-AKT signaling pathway. (**a**) MA plots illustrating differentially expressed genes (FDR < 0.05, |logFC| > 1, red dots) between conditionally expressed OVTOKO cells with (*n* = 3) and without DOX (*n* = 3). The overexpression of *PRELP* is shown as green dots. The average logCPM is plotted on the x-axis. The logFC is plotted on the y-axis. (**b**) KEGG pathway enrichment analysis of differentially expressed genes in OVTOKO cells that overexpressed PRELP. Dot plot showing GeneRatio on the x-axis and terms sorted by GeneRatio on the y-axis. The *p*-value is displayed as a gradient from red to blue. The number of counts is indicated by the size of the black circle. (**c**) Volcano plots illustrating differentially expressed genes (FDR < 0.05, |logFC| > 1, red dots) between the same set of OVTOKO cells as (**a**). Differences in Log_2_ fold change in gene expression values are plotted on the x-axis. Adjusted *p*-values calculated using the Benjamin–Hochberg method are plotted on the y-axis. Genes corresponding to the PI3K-AKT signaling pathway are shown as large red circles.

If PRELP affects the ligand-mediated pathways, fluctuations in several relevant genes should be observed. To this end, we performed RNA-seq on conditionally PRELP-expressing cells derived from the OVTOKO and SKOV3 cell lines. Indeed, we found that more than 1000 genes were significantly altered by PRELP expression (Figure 5a and Supplementary Tables S5 and S6), indicating that PRELP is a regulator of various genes. Furthermore, many genes associated with the PI3K-AKT signaling pathway were altered by PRELP expression and ranked at the top in the KEGG pathway enrichment analysis (Figure 5b,c and Supplementary Table S9). A similar trend was observed in SKOV3 cells that overexpressed PRELP (Supplementary Figure S3 and Supplementary Tables S7, S8 and S10). These results suggest that PRELP regulates the PI3K-AKT signaling pathway, which might have inhibitory effects on cell proliferation.

## 4. Discussion

In this study, we provided a novel perspective on the molecular features of the pathogenesis of OCCC. We first showed that the *PRELP* gene expression is repressed using public datasets of clinical tissues from patients with ovarian cancer compared with normal tissues. Furthermore, all OCCC cell lines we tested exhibited a significant downregulation of the *PRELP* expression compared with ovarian endometriotic epithelial cells and the putative progenitor of OCCC, which reflects that *PRELP* repression may be involved in early OCCC tumorigenesis. Thus, studying *PRELP* as a biomarker for the early detection of OCCC is intriguing.

How is *PRELP* expression suppressed during OCCC tumorigenesis? Deletions and somatic mutations in the *PRELP* gene are relatively rare (Figure 1), making it unlikely that genetic alterations are the primary cause of the suppression of *PRELP* expression. Indeed, ChIP-seq analyses revealed that using normal ovary and OCCC tissues and transcriptionally active marks, such as H3K4me3 and H3K27ac, were diminished at the *PRELP* gene promoter region in OCCC compared with those in normal ovary tissues, indicating that the *PRELP* gene is repressed, at least in part, by an epigenetic mechanism.

Epigenetic mechanisms have been shown to contribute profoundly to OCCC tumorigenesis. For example, mutations in the AT–rich interaction domain 1A (*ARID1A*) and phosphatidylinositol-4,5-bisphosphate 3-kinase (PIK3) catalytic subunit alpha (*PIK3CA*) have been frequently detected in OCCC cases [31,32,33]. *ARID1A* encodes the BAF250 protein as a subunit of the switch/sucrose non-fermentable chromatin–remodeling complex that facilitates the epigenetic regulation of the chromatin structure and transcription factors [34,35]. BAF250 protein loss can be detected in atypical endometriosis but not in distant endometriotic lesions [34]. *ARID1A* and *PIK3CA* mutations may contribute to OCCC tumorigenesis through sustained interleukin (IL)-6 production [35]. Moreover, Yano et al. showed that histone deacetylases, HDAC6 and HDAC7, are more strongly expressed in OCCC than in other subtypes of ovarian cancer [36]. How these epigenetic alterations and *PRELP* suppression contribute to OCCC tumorigenesis are currently unclear; however, one scenario is that the interplay between ARID1A and HDACs could significantly contribute to OCCC tumorigenesis [37]. Therefore, it is possible that the dysregulation of HDACs directly suppresses *PRELP* gene expression.

Using conditionally, PRELP-expressing cells derived from OCCC cell lines in which the ARID1A protein expression was repressed [38], we revealed that PRELP significantly altered the gene expression of more than 1000 genes. Particularly, it regulates a set of genes related to the PI3K-AKT signaling pathway (Figure 5). How PRELP can alter these pathways is unclear, even though it is a secreted ECM protein. However, recent proteomic studies have suggested that PRELP interacts with two growth factor receptors: insulin-like growth factor I receptor and low-affinity nerve growth factor receptor (p75NTR) [39], suggesting that PRELP functions as a ligand. Although many questions remain unanswered, elucidating the subcellular localization of PRELP may provide clues to the answers to these questions.

Although the aforementioned results provide the unexpected finding that PRELP is not only a connective tissue-anchored molecule but also a tumor-suppressive signaling molecule in OCCC, many unresolved issues remain. For example, experiments, such as the cell-cycle analysis, are needed to elucidate more detailed molecular mechanisms. In future studies, we plan to comprehensively analyze the protein expression level of a group of genes involved in the PI3K-AKT signaling pathway with or without PRELP using immunoblotting. Moreover, the anticancer mechanisms of the PRELP protein in OCCC have not been elucidated. The xenograft mouse model in the OCCC cell line is one of the next studies we wish to conduct for further validation of the function of PRELP. Lastly, due to the limited number of clinical samples used in this study, conducting clinical studies is impossible, such as validating a biomarker. Most of the cohort databases of ovarian cancer correspond to HGSOC patients, and there is no big dataset of OCCC patients. Several PI3K/AKT/mammalian target rapamycin (mTOR) pathway inhibitors have been used in clinical trials for OCCC [40]. To validate the clinical function of PRELP, we have to perform a prospective study to establish a dataset of OCCC patients for genetic and epigenetic analyses.

Although we analyzed pathways at the induction of PRELP expression, specifically in OCCC, there have been some studies reporting PRELP in other cancer types. HDAC inhibitors show anti-cancer effects by partially regulating the function of PRELP in bladder cancer. Furthermore, the acetylation of lysine residue 5 of histone H2B in the *PRELP* gene promoter region is a marker for the restoration of PRELP expression [23]. The overexpression of PRELP correlates with better patient survival and inhibits both cell proliferation and migration in hepatocellular carcinoma [41]. A peptide corresponding to the N-terminal heparin-binding domain of PRELP inhibits osteoclastogenesis in breast cancer metastases [42]. Thus, PRELP may regulate a set of genes related to the PI3K-AKT signaling pathway regardless of carcinoma type to some extent. However, no single data type, such as somatic mutations or gene expression, can capture the complexity of all the factors relevant to understanding phenomena such as cancer. Recently, integrated genetic and epigenetic dataset analyses using machine learning algorithms have emerged [43]. Our future directions may focus on epigenetics combined with RNA-seq and ChIP-seq, which may become part of the data to predict clinical outcomes. Elucidating the roles of PRELP in cancer may help understand the clinical significance of the classified genes identified by machine learning algorithms.

## 5. Conclusions

This study demonstrated that the *PRELP* gene was broadly repressed in OCCC, which was mediated by epigenetic mechanisms involving the loss of active histone marks. Furthermore, induced PRELP expression in OCCC cell lines inhibited cell proliferation, presumably via the PI3K-AKT signaling pathway. These findings suggest that PRELP is not only a molecule anchored in the connective tissues but is also a signaling molecule that acts in a tumor-suppressive manner in OCCC. Further investigation of the molecular mechanisms underlying OCCC tumorigenesis can serve as the basis for early detection and novel therapeutic strategies for OCCC toward precision medicines.

## Data Availability

Raw data for RNA-seq have been deposited in the DNA Data Bank of Japan with DRA014790. Raw data from ChIP-seq using human-derived tissue samples will not be disclosed at this time for ethical reasons. All codes in this study are available upon request.

## Supplementary Materials

The following supporting information can be downloaded at https://www.mdpi.com/article/10.3390/jpm12121999/s1. Figure S1: Soft agar colony formation assay. Figure S2: H3K27me3 marks in the PRELP gene locus. Figure S3: MA plots, KEGG pathway enrichment analysis, and Volcano plots in SKOV3. Table S1: Clinical sample characteristics. Table S2: PRELP mRNA expression and ovarian tissue types. Table S3: PRELP mRNA expression and CNAs. Table S4: PRELP mRNA expression and somatic mutations. Table S5: Upregulated genes upon PRELP overexpression in OVTOKO. Table S6: Downregulated genes upon PRELP overexpression in OVTOKO. Table S7: Upregulated genes upon PRELP overexpression in SKOV3. Table S8: Downregulated genes upon PRELP overexpression in SKOV3. Table S9: The KEGG pathway in PRELP-overexpressed OVTOKO. Table S10: The KEGG pathway in PRELP-overexpressed SKOV3. Table S11: Information on certified cell lines. Raw Data S1: Uncropped immunoblot data are shown in Figure 3.
