# Supplementary material for: Tumor Suppressive Role of the PRELP Gene in Ovarian Clear Cell Carcinoma
Source: J Pers Med. 2022 Dec 2;12(12):1999. doi: 10.3390/jpm12121999 (PMC9785654; doi:10.3390/jpm12121999)
Supplement: Supplementary file 1 [file jpm-12-01999-s001.zip › Supplementary_materials_legends_revised.docx]

**Supplementary Figures, Tables, and Raw Data**


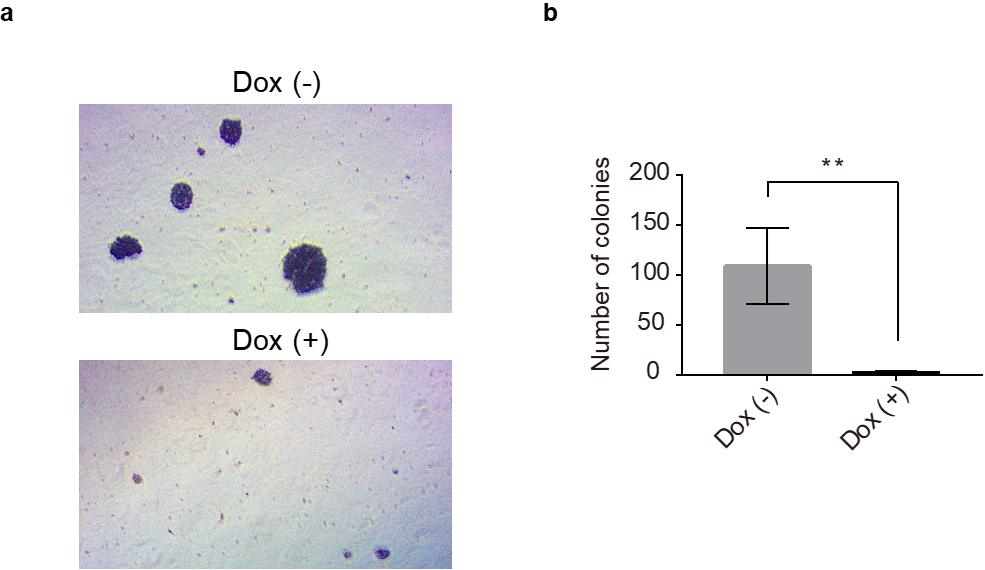


**Supplementary Figure S1.** PRELP overexpression inhibits cell proliferation in ovarian cancer cell lines. Soft agar colony formation assay with DOX treatment in *PRELP*-inducible SKOV3. (**a**) Representative images of colonies are shown. White bars indicate 100 μm. (**b**) The number of colonies is shown. Error bars represent mean ± standard deviation of three biological replicates. Note that colonies are counted when their size is ≥ 2,500 μm^2^. Statistical analysis was performed using Student’s t-test. ***P* < 0.01.

**
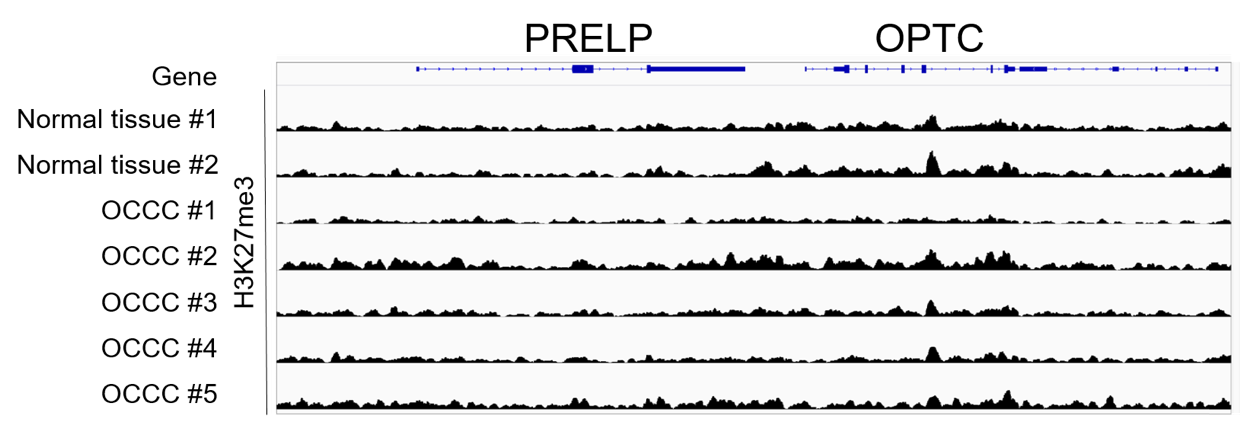
**

**Supplementary Figure S2.** Comparison of H3K27me3 marks in the *PRELP* gene locus between normal tissues and OCCCs. Illustrated as in Figure 5, except for IGV tracks of H3K27me3 ChIP-seq are shown. All data ranges are standardized as 0–2.


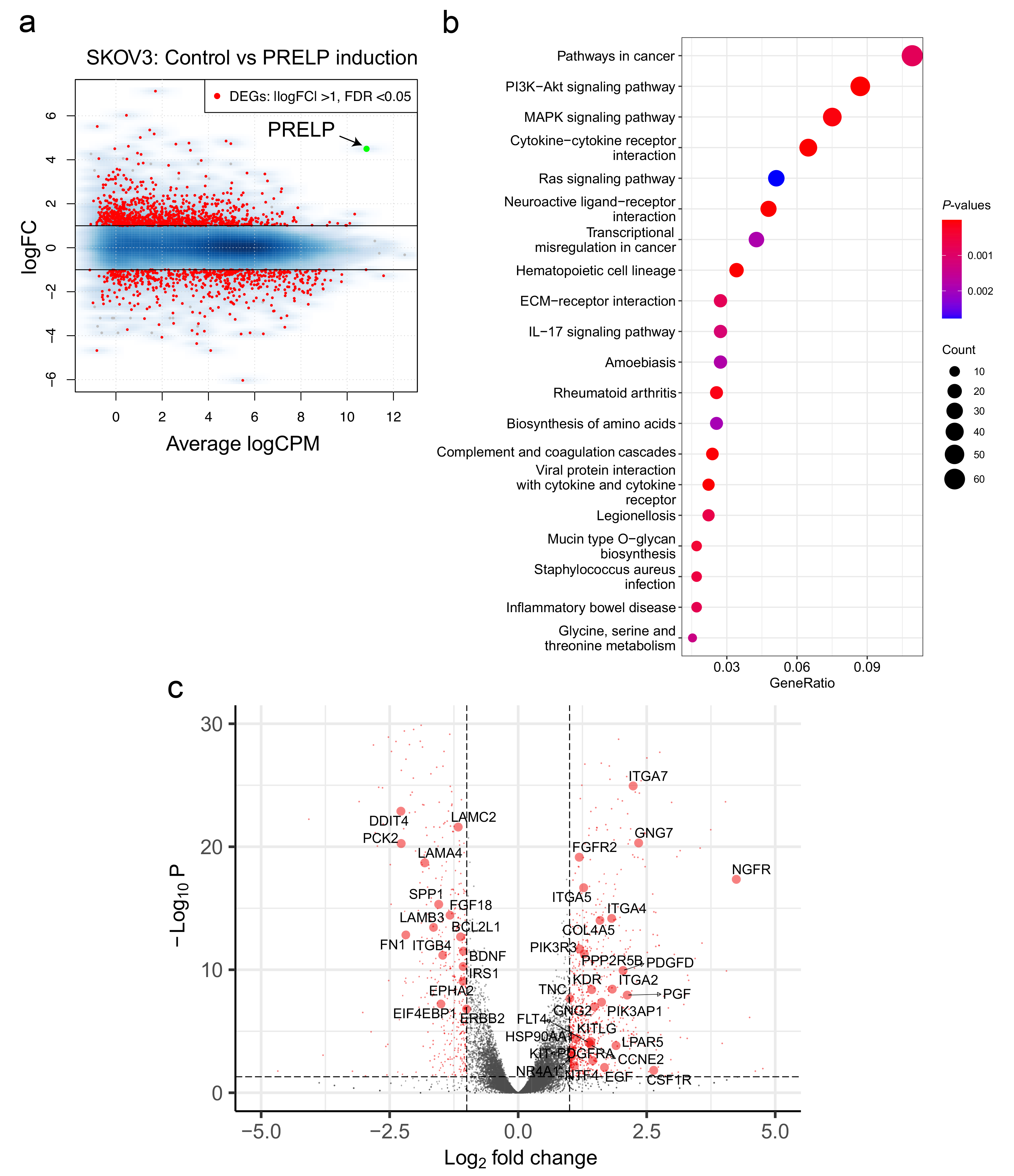


**Supplementary Figure S3.** PRELP regulates the PI3K-Akt signaling pathway in SKOV3. Plotted as in Figure 5, except that another cell line, SKOV3, was used.

**The file for supplementary tables contains the following materials.**

**Supplementary Table S1.** Clinical sample characteristics.

**Supplementary Table S2.** The individual data for the correlation between *PRELP* mRNA expression and ovarian tissue types.

**Supplementary Table S3.** The individual data for the correlation between *PRELP* mRNA expression and copy number aberrations (CNAs).

**Supplementary Table S4.** The individual data for the correlation between *PRELP* mRNA expression and somatic mutations.

**Supplementary Table S5.** The list of upregulated genes upon PRELP overexpression in OVTOKO cells (log_2_ FC >1, FDR < 0.05, n = 1,180).

**Supplementary Table S6.** The list of down-regulated genes upon PRELP overexpression in OVTOKO cells (log_2_ FC > 1, FDR < 0.05, n = 584).

**Supplementary Table S7.** The list of upregulated genes upon PRELP overexpression in SKOV3 cells (log_2_ FC > 1, FDR < 0.05, n = 948).

**Supplementary Table S8.** The list of down-regulated genes upon PRELP overexpression in SKOV3 cells (log_2_ FC > 1, FDR < 0.05, n = 598).

**Supplementary Table S9.** The list of KEGG pathway of differentially expressed genes in PRELP-overexpressed OVTOKO cells.

**Supplementary Table S10.** The list of KEGG pathway of differentially expressed genes in PRELP-overexpressed SKOV3 cells.

**Supplementary Table S11.** Information about the certified cell lines. STR method was used for certification.


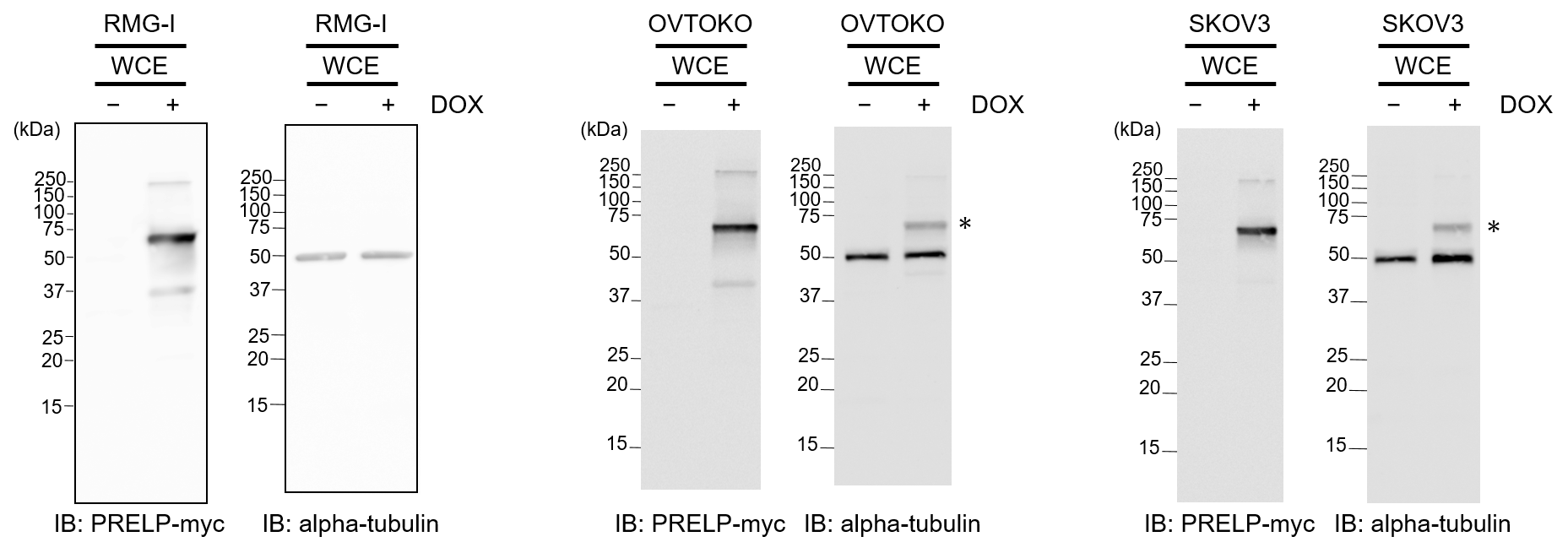


**Supplementary Raw Data S1.** Uncropped immunoblot data shown in Figure 3. Asterisks indicate insufficiently stripped PRELP-myc.
