# Supplementary figures and images for: Tumor Suppressive Role of the PRELP Gene in Ovarian Clear Cell Carcinoma
Source: J Pers Med. 2022 Dec 2;12(12):1999. doi: 10.3390/jpm12121999 (PMC9785654; doi:10.3390/jpm12121999)

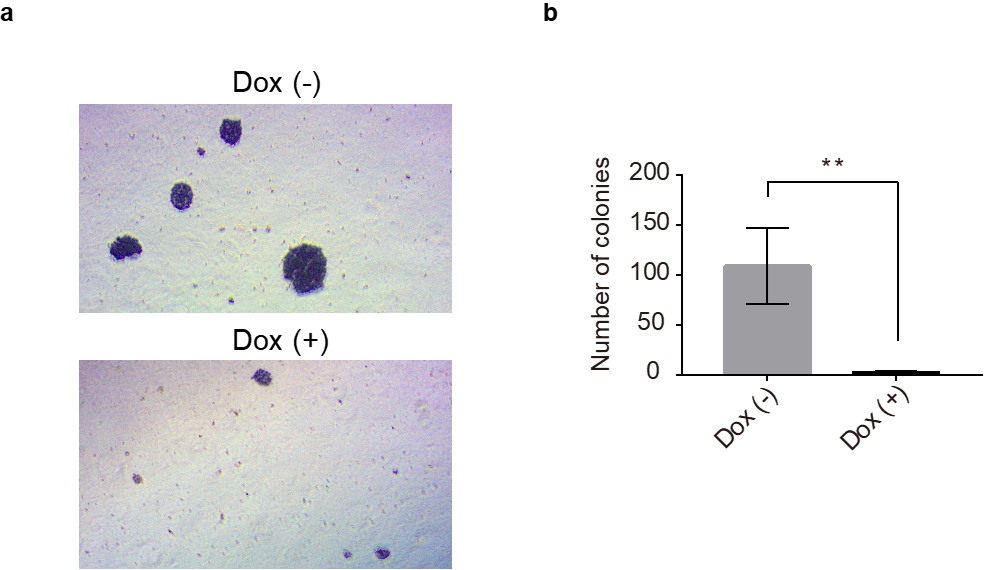

Supplement: Supplementary file 1 [file jpm-12-01999-s001.zip › Supplementary Figure S1.png]

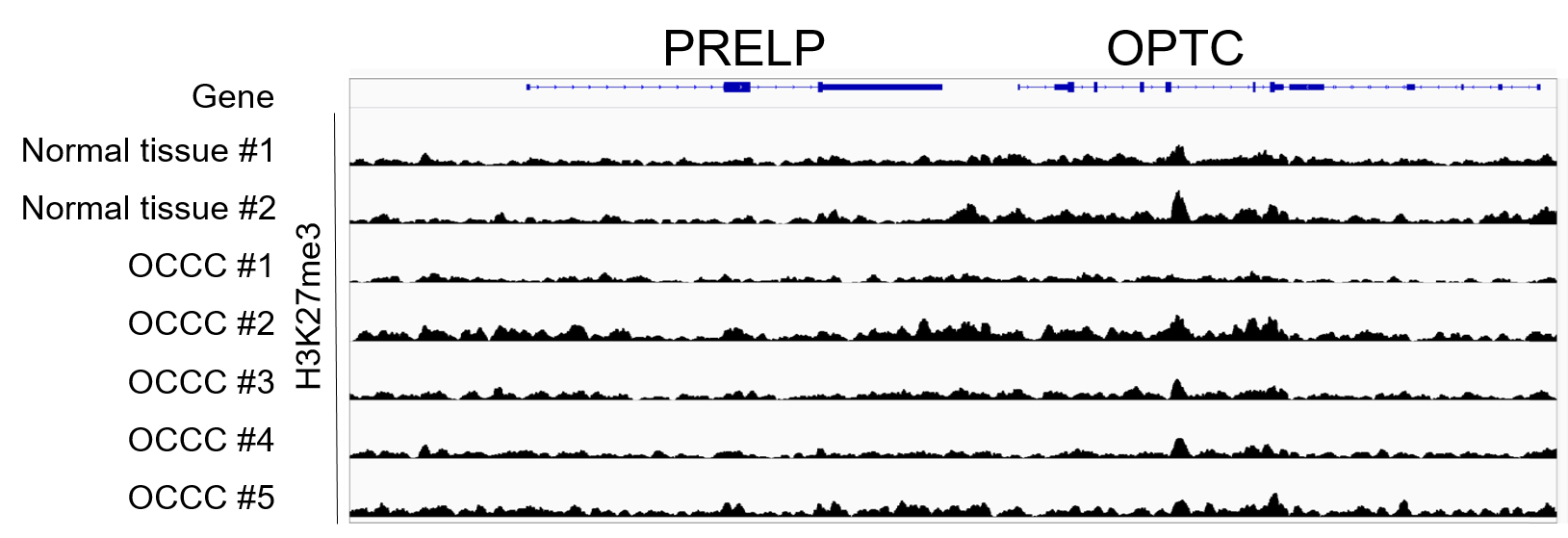

Supplement: Supplementary file 1 [file jpm-12-01999-s001.zip › Supplementary Figure S2.png]

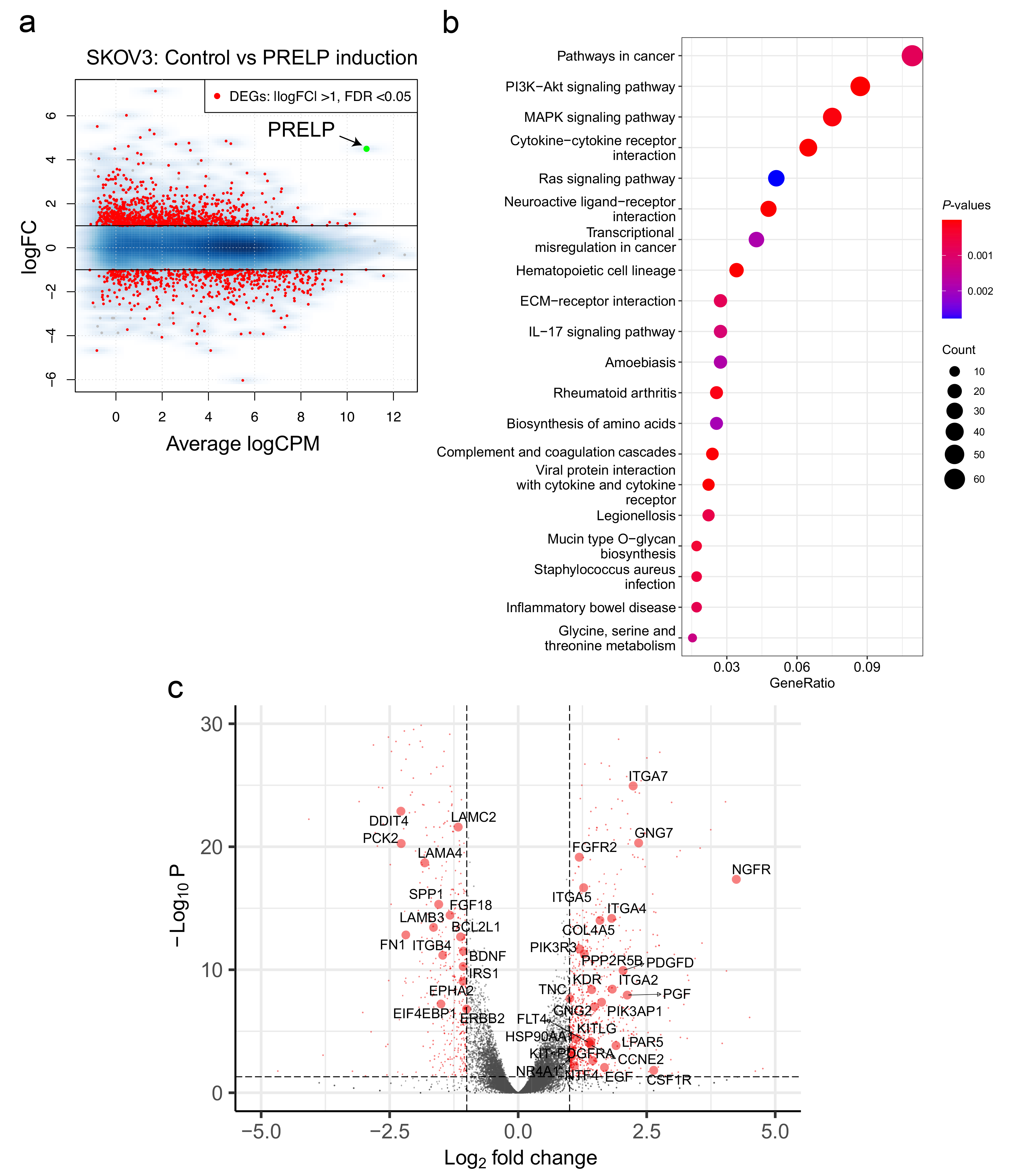

Supplement: Supplementary file 1 [file jpm-12-01999-s001.zip › Supplementary Figure S3_revised.png]

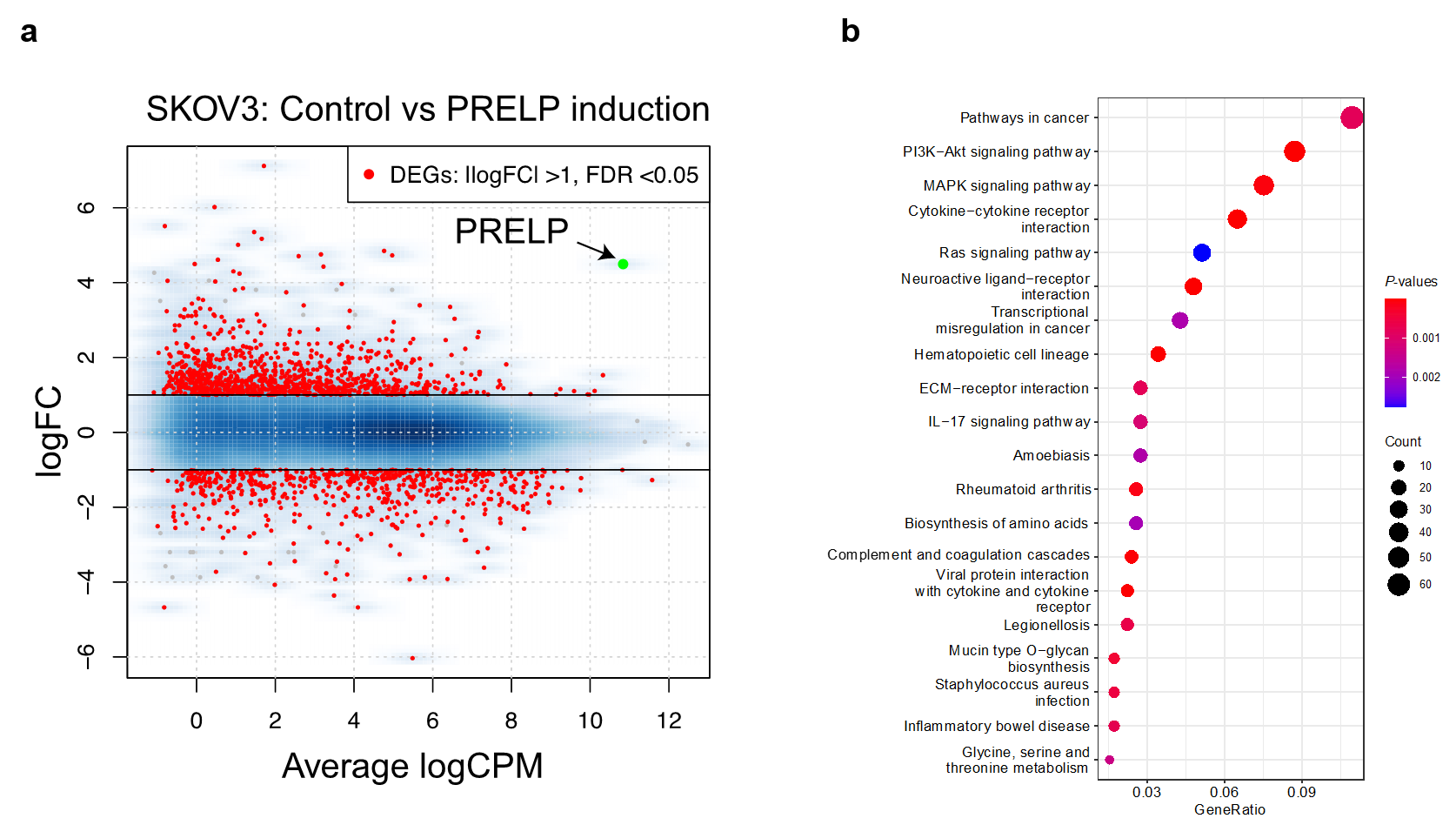

Supplement: Supplementary file 1 [file jpm-12-01999-s001.zip › Supplementary Figure S4.png]

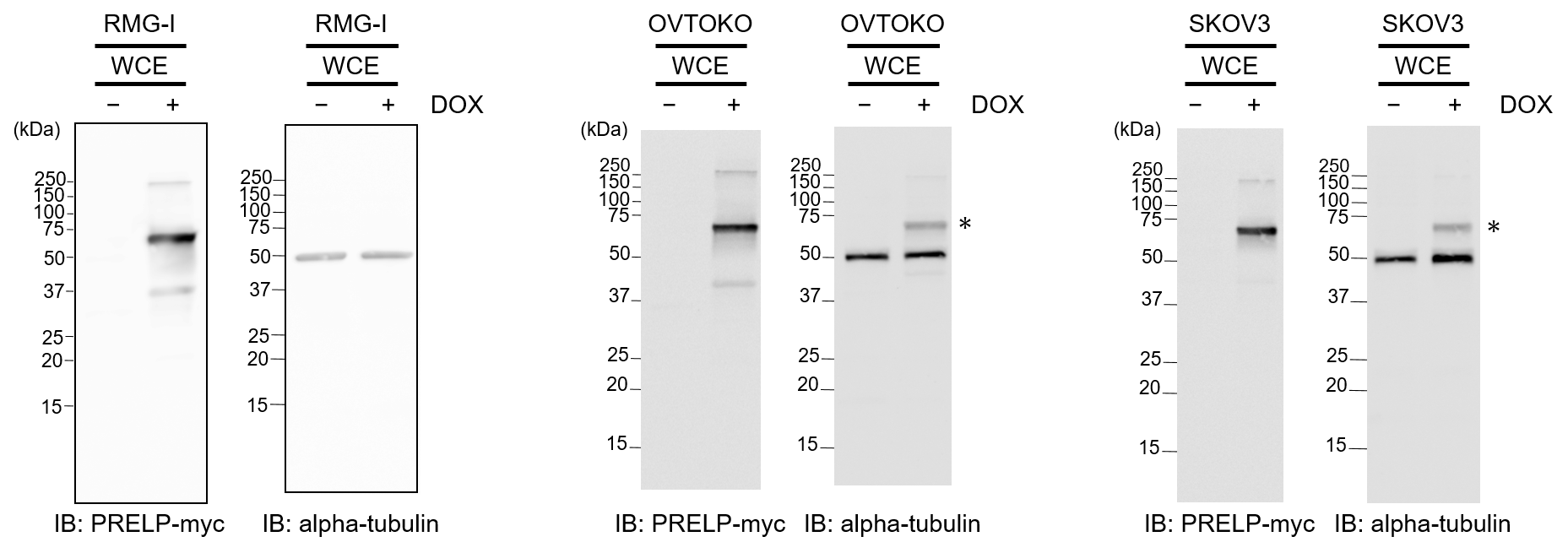

Supplement: Supplementary file 1 [file jpm-12-01999-s001.zip › Supplementary Raw Data S1.png]
